# Hidden role of gut microbiome in mental health

**DOI:** 10.1192/j.eurpsy.2022.1789

**Published:** 2022-09-01

**Authors:** A. Duarte, I. Simões, C. Cordeiro, P. Martins

**Affiliations:** 1 Centro Hospitalar Lisboa Norte - Hospital Santa Maria, Psychiatry And Mental Health, Lisboa, Portugal; 2 Centro Hospitalar Lisboa Norte, Psychiatry, Lisboa, Portugal

**Keywords:** microbiome, mental health, probiotics, psychobiotics

## Abstract

**Introduction:**

The recent literature indicates that the gut microbiota may affect brain functions through endocrine and metabolic pathways, antibody production and the enteric network while supporting its possible role in the onset and maintenance of several neuropsychiatric disorders, neurodevelopment and neurodegenerative disorders.

**Objectives:**

The aim of this work is to discuss the role of probiotics, prebiotics, or synbiotics as a potential treatment for symptoms of depression, anxiety, and stress.

**Methods:**

Pub Med database was searched using following key words: “probiotics”, “prebiotics”, “mental disorders”, “psychological disorders”.

**Results:**

Although the exact mechanism is unknown, there is a link between the gut and mood disorders. Psychosocial factors, such as quality of life or well-being, are greatly influenced by gut function and there is a strong correlation between psychosocial features and gastrointestinal disorders. Elevated stress, anxiety, and depression are linked to intestinal dysbiosis and mood disorders are disproportionately high in patients with functional gut disorders. So, psychobiotics may provide benefit when used in conjunction with current antidepressant medications. Probiotics may exert their therapeutic benefits by restoring microbial balance in the gut, and also by minimizing gastrointestinal complaints, allowing for the effects of antidepressant medication to not be reduced. Stress and immune responses were improved following psychobiotic intervention in stressed adults. Psychobiotics offer potential alternative treatment options in mood disorders and their accompanying symptoms.

**Conclusions:**

Pro and prebiotics can improve mental health and psychological function and can be offered as new medicines for common mental disorders. However, more clinical studies are required to support the clinical use of probiotics.

**Disclosure:**

No significant relationships.

